# Clinicopathologic patterns and treatment outcomes of pediatric classic hodgkin lymphoma in Ethiopian newly opened cancer treatment centers

**DOI:** 10.1186/s12887-026-06743-4

**Published:** 2026-03-25

**Authors:** Degalem Tilahun Worku, Diriba Fufa Hordofa, Yalew Melkamu Molla, Negasa Tesema Keneni, Zufan Yiheyis Abriham, Mulugeta Ayalew Yimer

**Affiliations:** 1 Department of Pediatrics and Child Health, School of Medicine, College of Medicine and Health Sciences, University of Gondar, Gondar, Ethiopia; 2 Department of Pediatrics and Child Health, School of Medicine, College of Medicine and Health Sciences, Jimma University, Jimma, Ethiopia; 3 Department of Medical Parasitology, School of Biomedical and Laboratory Sciences, College of Medicine and Health Sciences, University of Gondar, Gondar, Ethiopia

**Keywords:** Pediatric, Hodgkin lymphoma, Treatment outcome, Gondar, Jimma, Ethiopia

## Abstract

**Background:**

Pediatric Hodgkin lymphoma (PHL) is the most common lymphoreticular cancer in adolescents aged between 15-19 years. The survival of PHL is over 95% in developed countries with or without radiation therapy. Proper staging and risk stratification of PHL before initiation of treatment is fundamental for cure. Although outcomes for PHL in developed countries are excellent, survival in low-income countries remains lower. PHL is one of the WHO six indexed cancers with a target survival of >60% by 2030 globally. The aim of the study was to evaluate clinicopathologic characteristics and treatment outcomes of PHL at two treatment centers in Ethiopia.

**Method:**

Retrospective cohort study was conducted from January 1,2020 to December 29, 2024 at both Gondar and Jimma pediatric treatment centers. All children under 18 years of age were included in the study population. Data were extracted from each patient’s medical chart review and entered with Epi-info to be analyzed using SPSS version 26. Overall survival (OS) and event free survival (EFS) were calculated and analyzed using Kaplan-Meier estimates. P-value less than 0.05 was considered statistically significant.

**Results:**

The study included 70 pediatric HL patients. The median age was 8.5 years (range: 4-18 years) and 77.1% of patients were males. Cervical lymphadenopathy was the commonest clinical presentation. Nodular sclerosis was the commonest histology subtype (34.3%) followed by mixed cellularity while 27% had uncategorized histology. Overall, 54.3% of patients had localized (stage I and II) and 45.7% of patients had advanced disease (stage III and IV) at initial staging. Adriamycin, Bleomycin, Vincristine, Etoposide, Prednisolone and Cyclophosphamide (ABVE-PC) was the commonest chemotherapy regimen used in our cohort (61.4%). Only 10% (7) of patients received radiation therapy all from the intermediate to high-risk group. In our study, the 3-year EFS and OS rate of pediatric HL was 85.7% and 98.6% accounting abandonment as censored. Treatment abandonment remains the challenge for cure in our cohort accounting about 31% to be censored.

**Conclusions:**

Our study showed that there was a trend towards earlier age at diagnosis of pediatric HL consistent with data from other developing countries. The treatment outcomes were good enough for newly opened centers in low-income countries with almost omission of radiotherapy, but treatment abandonment remains the challenge for cure as well affects the survival analysis in our cohorts. The treatment protocols used in this study surpassed the WHO indexed pediatric cancer survival target by 2030.

## Introduction

Pediatric HL is a mature B-cell lymphoma, defined histologically by the presence of large, bi/multinucleated cells called Hodgkin Reed Sternberg cells, the initial histological description dating back to 1832 by T. Hodgkin [1].

The prognosis of childhood cancer varies from complete cure to near-certain death, depending on which part of the world the child lives in [2–5]. The 5-year survival for childhood malignancies is typically between 80% and 90% in high-income countries (HICs), 20% to 30% in LMICs, and as low as 10% in certain East African nations with insufficient access to appropriate treatment [1, 6, 7].

The most frequent cancer in adolescents, PHL, is a malignant lymphoreticular transformation that peaks between the ages of 15 and 19 and makes up around 6% of childhood cancers. In roughly 70–80% of cases, PHL manifests as a painless, chronic, or gradually growing lymph node over the cervical or supraclavicular regions [6, 8, 9]. Reed Sternberg cells from excisional node biopsy and immunohistochemistry are confirmatory tests for pediatric HL [8–12]. Classic pediatric HL which includes four histologic variants is the commonest type of PHL histologically [11]. Immunohistochemistry will be positive for CD15 and CD30 along with EBER1/2 and EBV may be harbored in the RS cells [6, 13].

In high-income countries, remarkable advances in pediatric oncology have led to cure rates above 80%-90% [1, 6–9, 13–16]. This success has not been translated in low income countries, where survival of children with cancer remained close to 10–30% [17]. The resultant survival gap has been attributed to delayed presentation, treatment abandonment, treatment-related mortality, and a higher rate of relapse due to inequities of access for proper treatments [1, 6, 17]. Improving cure rates of PHL is one of the key cancer treatment measures of a nation/country stated by the WHO where survival is targeted to be above 60% in 2030.

In the modern era, treatment of PHL is based on risk stratification and response adapted approach, where the radiation therapy is avoided or reduced in doses [4, 18–20]. Unfortunately the 5 years survival of most childhood cancers including PHL in LMICs is not more than 30% due inequity of treatment access [17]. Assessing the treatment outcome of PHL in selected Ethiopian pediatrics cancer treatment centers can be used to assess a pediatric oncology center’s effectiveness in treating and surviving common pediatric cancers [17].This is the first study to lay ground about the treatment outcome of PHL in Ethiopia regarding the treatment outcomes of PHL. Therefore, this study aims to assess the clinicopathologic characteristics and treatment outcomes of PHL at the University of Gondar and Jimma pediatric cancer treatment centers since there are only few studies regarding the treatment outcomes of PHL in Low-income settings particularly in Ethiopia.

## Methods and Materials

### Study design, area, period

A hospital based retrospective cohort study was conducted on patients treated for PHL from January 1, 2020 to December 29, 2024. Ethical clearance obtained from University of Gondar College of Medicine and health science after proposal was submitted and reviewed by the ethical committee.

Data were collected from both Gondar and Jimma pediatrics cancer treatment centers through chart review. There are only 5 pediatrics cancer treatment centers in Ethiopia where Gondar and Jimma are of the two centers. Each center has only one consultant Oncologist while both centers are dedicated for about 30 million to 40 million catchment areas. Annually about 300 childhood cancer cases are diagnosed in both centers. There was no radiotherapy at Gondar pediatric cancer treatment but Jimma center. All children under 18 years, confirmed histologically and treated for PHL at both centers were included by census method.

### Study population

The study population were those children treated for PHL at Gondar and Jimma pediatric cancer treatment centers during the study period.

### Inclusion and exclusion criteria

All children under 18 years confirmed histologically for PHL and treated at Gondar and Jimma pediatric cancer centers were included while those children with incomplete data on both clinical and histological characteristics were excluded.

### Sample size and sampling procedure

All children treated for PHL from both treatment centers were included in the study using census method and accordingly 70 study participants met inclusion criteria.

### Study variables

Treatment outcomes of PHL were the dependent variables while treatment modalities, clinical characteristics of the study participants, histology type of the disease and chemotherapy regimens were independent variables in this study.

### Operational definitions

#### Treatment outcome

refers to the end points of treatment as abandonment, complete response, partial response, progressive disease, relapsed disease or death.

#### Intermediate/high risk

Refers to the risk strata of the case based on the Children’s Oncology Group (COG) assignment criteria.

#### Low risk

Refer to category of cases stratified based COG criteria.

#### Abandonment

Treatment interruption for more than 28 days or unable to complete the planned treatment schedule.

### Data collection instruments and procedures

Data were collected from registration logbooks and patient’s medical chart retrospectively. The data were collected by structured questionaries for medical record review developed for this study. The data were collected by two pediatric Hematology oncology General practitioners after 2 days of formal training. The final status of each patient collected from the date of diagnosis to the last date of visited documented from the medical charts. The chart /protocol of treatment reviewed in detail for death certificates, progress notes and date of declared relapse/progressed disease. The data collectors were continuously supervised by the principal investigator during the data collection process.

### Data analysis and interpretations

The data collected were checked for completeness and consistency by the investigator. The collected data were cleaned, coded, and entered Epi- Info version 7 and then exported into SPSS Version 26.0 for analysis. Descriptive statistics using frequencies and percentages for categorical variables and means/median and SD for quantitative variables were used. Censoring was defined as the last day patient’s status was documented on hospital visit either for scheduled follow up or treatment dates. The study’s OS and EFS for PHL cases were computed using the Kaplan-Meier technique. *P*-values less than 0.05 were considered statistically significant.

## Results

**Fig. 1:**
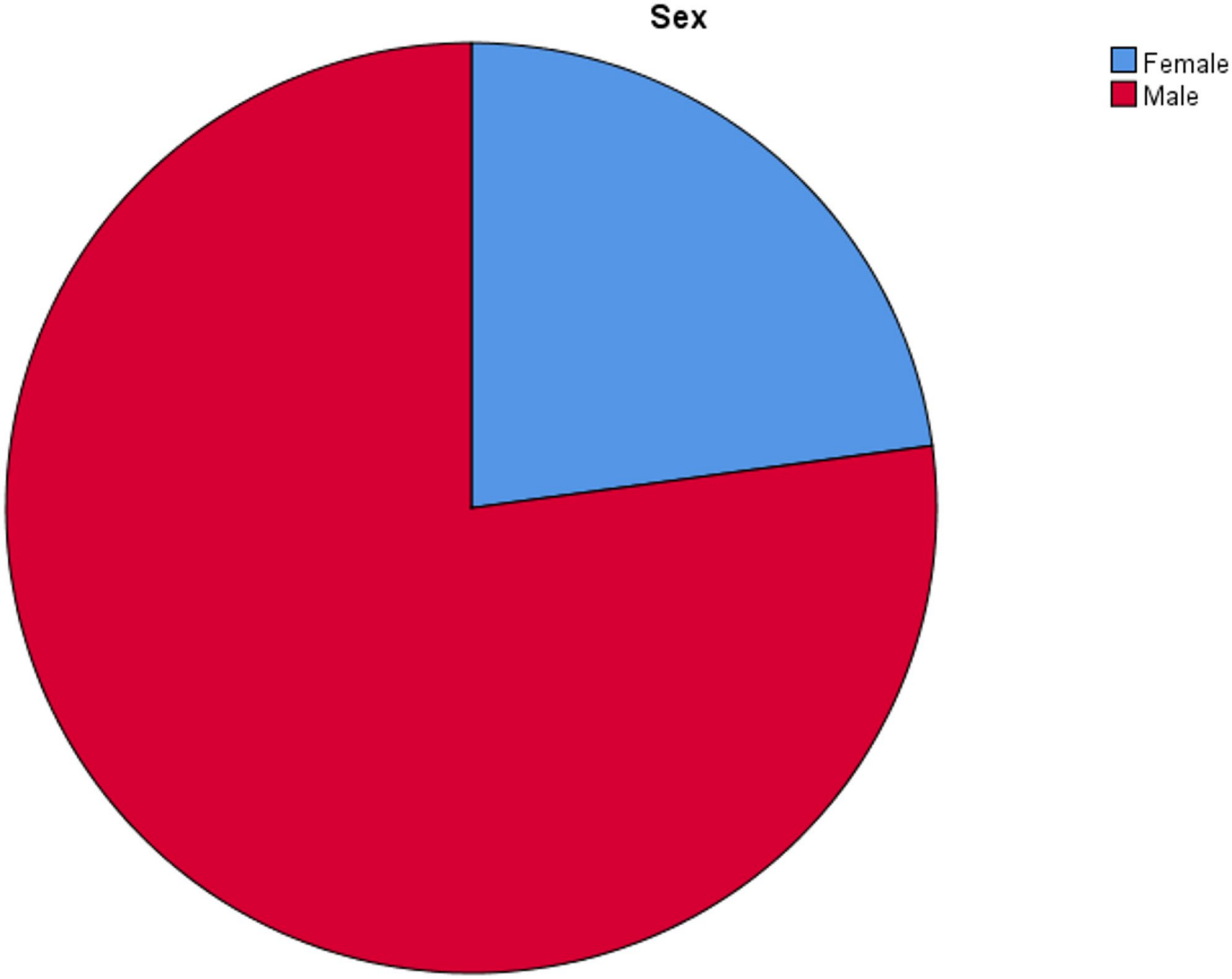
Sex distribution of pHL participants in the study

During the study period, between January 2020 to December 2024, 70 pediatric patients with PHL fulfilled the inclusion criteria from the Pediatric Oncology units of Gondar and Jimma pediatric cancer treatment centers. Age at diagnosis ranged between 4 and 18 years with a median of 8.5 years. The most affected age groups in this cohort were children between 5 and 10 years, accounting for 62.9% and 12.9% of the cohort were aged under 5 years. About three fourth (77.1%) were males with male to female ration of 3.4:1 (Fig. 1) (Table 1).

**Table 1:** Clinical and demographic Characteristic of the Patients

Characteristics of Patients	No.	%
Sex		
Male	54	77.1
Female	16	22.9
Age, years		
< 5	9	12.9
> 5–10	44	62.9
> 10	17	24.3
Stage of the disease		
Stage I	10	14.3
Stage II	28	40
Stage III	23	32.9
Stage IV	9	12.9
B-symptoms		
Yes	24	34.3
No	46	65.7
c- HL subtypes		
Mixed cellularity (MC)	15	21.4
Nodular sclerosis (NS)	24	34.3
Lymphocyte-rich (LR)	10	14.3
Lymphocyte-depletion (LD)	2	2.9
Uncategorized	19	27.1
WBC at diagnosis		
< 4 × 10^3^/µl	8	11.4
4–15 × 10^3^/µl	60	85.7
> 15 × 10^3^/µl	2	2.9
PLT at diagnosis		
< 100 × 10^3^/µl	3	4.3
100–450 × 10^3^/µl	51	72.9
> 450 × 10^3^/µl	16	22.9
Therapeutic modalities		
ABVE-PC	43	61.4
ABVD	27	38.6
Relapsed disease		
Yes	10	14.3
No	60	85.7
Radiation therapy		
Yes	7	10
No	63	90

*ABVE* Adriamycin, Bleomycin, Vincristine and Etoposide, *ABVD* Adriamycin, Bleomycin, Vincristine and Dacarbazine, *PC* Prednisolone and Cyclophosphamide, *wbc* white blood cells, *PLT * Platelets, *c-HL * classic Hodgkin Lymphoma

**Fig. 2:**
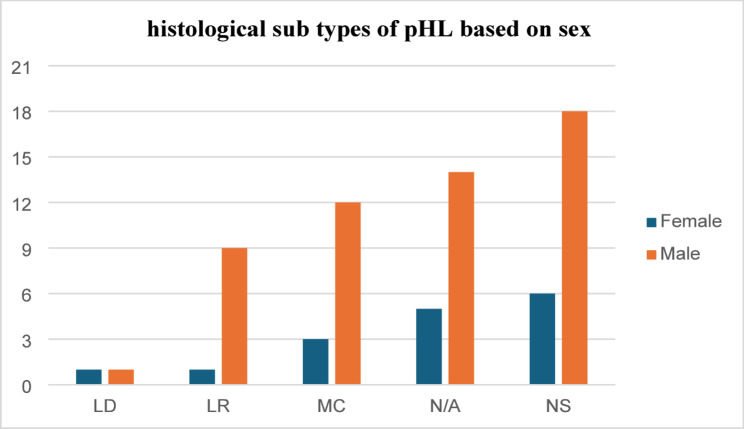
Histologic subtypes of classic-PHL based on sex of the Cohort. LD= lymphocyte-depletion; MC= mixed cellularity; LR= lymphocyte rich; NS=nodular sclerosis; N/A= uncategorized

In our cohort 20% [14] of patients presented with symptoms longer than 12 months while 48.6% [34] had symptoms longer than 6 months before presentation. In addition, the clinical symptoms ranged as short as 2 weeks to as long as 36 months before diagnosis was made. HIV results were available for all cases and only 1.4% [1] patient was positive. One third of the cases (34.3%) had at least one B-symptom at presentation. According to the Ann Arbor staging system, 54.3% of patients had localized (stage I and II) while 45.7% had advanced (stage III and IV) disease. Nodular sclerosis followed by Mixed cellularity were the commonest histologic patterns representing 34.3% and 21.4% respectively while 27.1% of the cases had unclassified classic PHL. Both of histologic types were common in male. (Fig. 2). Mixed cellularity was common in under five while nodular sclerosis was common in children above 5 years of age. Nineteen (27.1%) of the cases were diagnosed based on Fine needle aspiration cytology (FNAC) and immunohistochemistry was done only for one case (1.4%) (Table 1).

Treatment was planned based on risk stratifications as 6 cycles of ABVD regimen for low- risk group and 8 cycles of ABVE-PC for intermediate to high -risk groups with or without radiotherapy. Interim assessment through imaging was done to decide the need of radiotherapy. Majority of patients 61.4% [43] were treated with ABVE-PC. Among patients treated with ABVE-PC, 16.3% [7] patients received radiation therapy after interim assessment. With related to relapse status 14.3% [10] of patients experienced relapsed disease (Table 1).

About 60% [42] cases presented cervical node involvement while mediastinal involvement was noted in 60% [42] of the cases. Inguinal node enlargement was noted in 8.6% [6] of the cases while spleen was involved in 18.6% [13] detected radiologically either as splenic enlargement or nodules. Majority of cases were confirmed by biopsy 65.7% [46] and 32.9% [23] by FNAC. (Table 2).

**Table 2:** Additional clinical characteristics of the Patients

Characteristics of Patients	No.	%
Systems involved at presentation on physical examination only		
Cervical node	42	60
Inguinal node	6	8.6
Spleen	5	7.1
Supraclavicular node	4	5.7
Mode of Dx		
Bx	51	72.9
FNAC	19	27.1
Immunohistochemistry	1	1.4
Mediastinal mass on CT-Scan		
Yes	42	60
No	28	40
Abdomen CT-scan		
Normal	40	57.1
Focal/diffuse spleen involvement	13	18.6
Nodes	7	10
Others (Spleen, Liver, Ascites, kidney nodules)	10	14.2
Risk classification		
IR/HR	43	61.4
LR	27	38.6
Bone marrow aspiration (BMA)		
N/A	63	90
Negative	7	10

*Bx* biopsy, *IHC * Immunohistochemistry, * FNAC * Fine Needle Aspiration Cytology, *IR * intermediate risk, *LR*   low risk, *HR* high risk, *N/A *  Not Analyzed

About 61.4% [43] were stratified as intermediate to high-risk group while 38.9% [27] had low risk stratification treatment group. In this cohort, bone marrow aspirate with biopsy were indicated only for 10% [7] patients and all were negative (Table 2).

In this cohort, 68.6% (48) patients completed their treatment, while 31.4% [22] patients abandoned their treatment during their active treatment phases. In this cohort, 50% [35] were alive free of the disease and 48.6% [34] alive with active disease following relapse or with the progressive status while 1.4% [1] died from high- risk groups after completing his first cycle of chemotherapy (Table 3).

**Table 3:** Patient final status, events and treatment outcome result

Characteristics of Patients	No.	%
Treatment outcomes		
Complete response	43	61.4
Abandoned	22	31.5
Progressive disease	5	7.1
Final status of the patients		
Alive free of disease	35	50
Alive with disease	34	48.6
Death	1	1.4
Duration of symptoms before diagnosis		
<=6months	36	51.4
7-12months	20	28.6
> 12months	14	20
HIV status		
Positive	1	1.4
Negative	69	98.6

### Survival outcomes

**Fig. 3:**
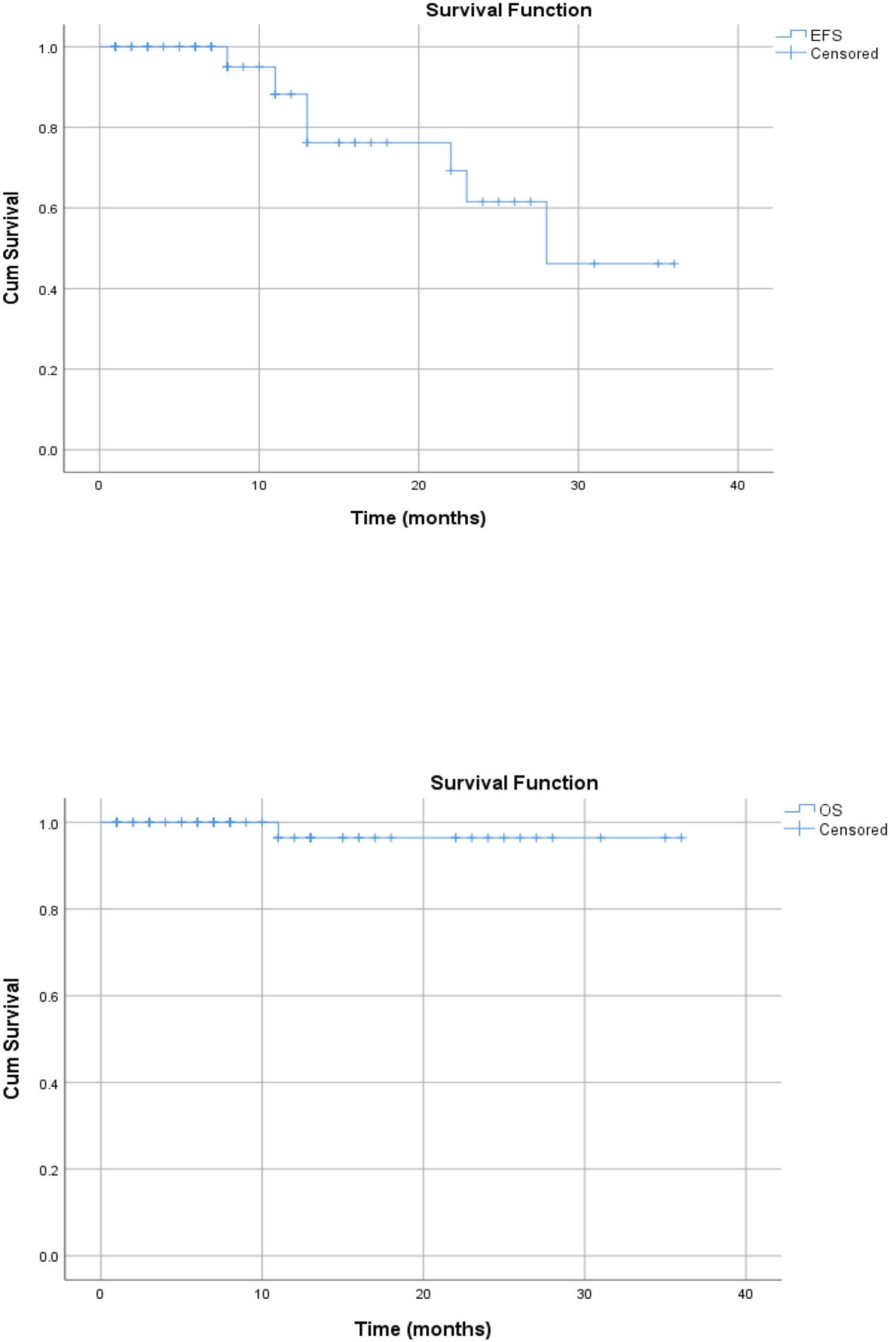
EFS and OS for the whole study group

The Median follow-up time was 28 months (range: 1–36) from the days of treatment initiations. Among relapses, 60% [6] patients relapsed within 1 year of diagnosis while, 4(40%) relapsed after 2 years of diagnosis. Most relapses, 7 (70%) were seen in stage II disease and 20% in stage IV disease. The most common histologic subtype relapsed was nodular sclerosis accounting for 50% relapses. The 3-years overall survival and Event free survivals for this cohort were 98.6% and 85.7% respectively (Fig. 3).

**Fig. 4:**
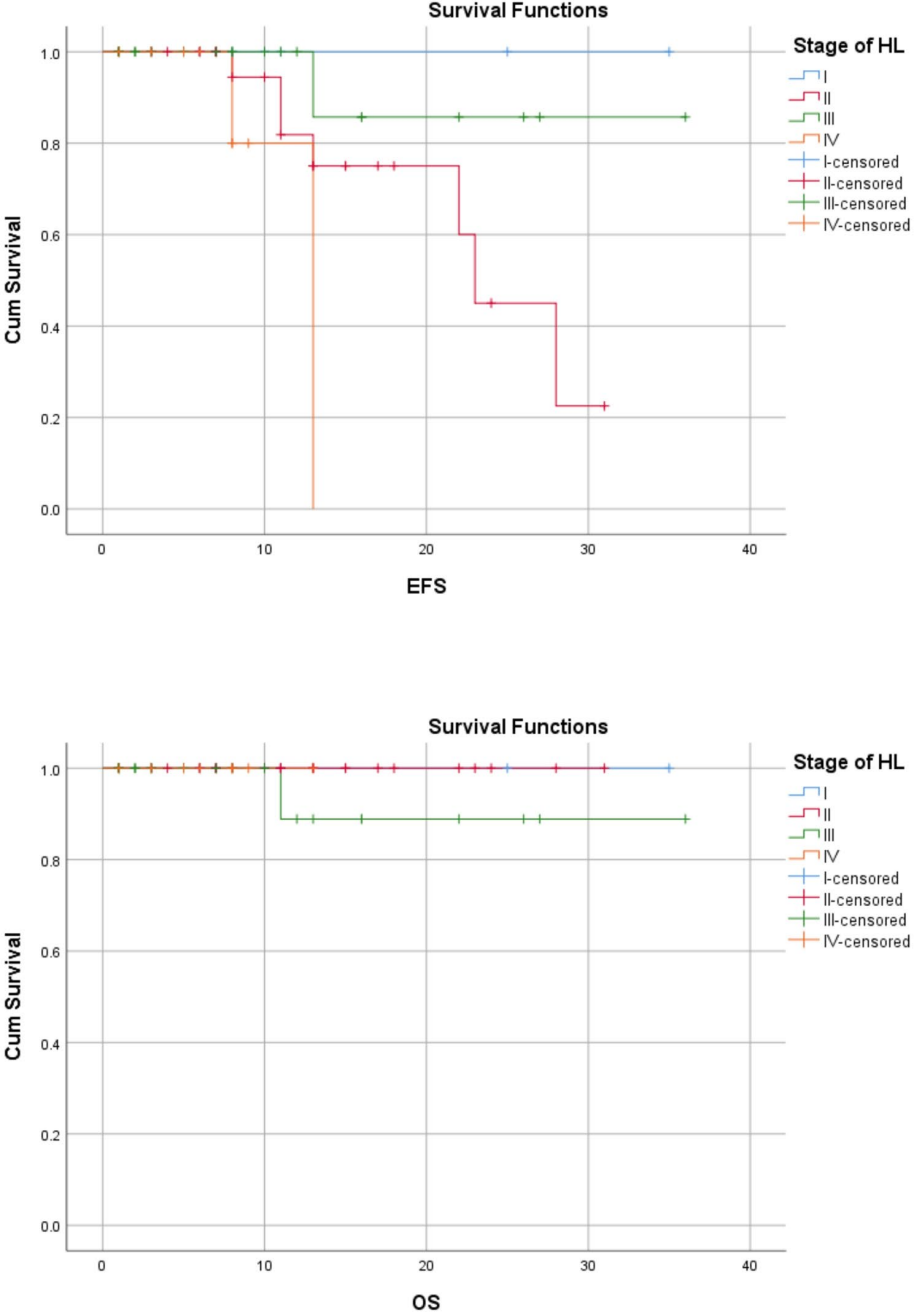
EFS and OS of HL stratified by clinical stage of disease

**Fig. 5:**
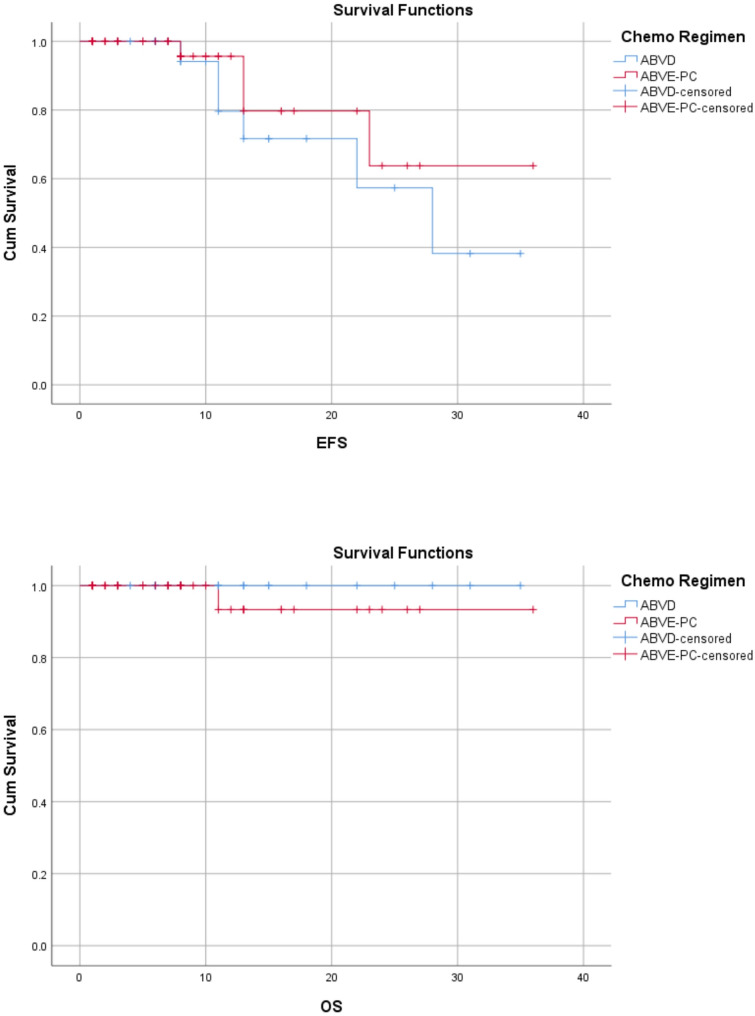
EFS and OS of HL stratified by chemotherapy regimen

In this study, the 3-year EFS for stages I, II, III and IV were 100%,75%,95.7% and 77.8% respectively. On the other hand, the OS for stages I, II, III and IV were 100%,100%, 95.7% and 100% respectively (Figs. 4 and Fig. 5). Stage of disease was statistically significant for EFS rates (log rank-test: *P* = 0.02) but was not true for the OS rates (log rank-test: (*P* = 0.55). Age, sex, histological subtype, risk strata, chemotherapy regimen were not statistically associated with outcome of treatment (Table 4).

**Table 4:** Results of Statistical Tests of Association Between OS and EFS and Study Variables in Hodgkin Lymphoma Patients

Variables	OS		EFS	
	Log rank	*P*	Log rank	*P*
Age	3.66	0.16	3.48	0.17
Sex	0.2	0.6	1.43	0.23
Stage of disease	1.51	0.55	28.5	02
Histological subtype	6	0.2	4.8	0.3
Risk classification	0.86	0.35	0.6	0.4
Chemotherapy regimen	0.86	0.35	0.6	0.4

## Discussion

Pediatric classic Hodgkin Lymphoma is a highly curable disease in the contemporary hematology-oncology protocols in the high-income countries, but it lagged behind in low-income settings like sub-Saharan regions. PHL age distribution and histopathologic subgroups differ among geographical, genetic, and environmental circumstances and treatment outcomes varies based on the regions [21, 22].

Our study described the epidemiological and clinical characteristics of 70 Ethiopian children with classic PHL who were treated at two pediatric Oncology treatment centers from Gondar and Jimma specialized hospitals, with 51.4% [36] from Gondar and 48.6 [34] were from Jimma pediatric cancer treatment centers. The ages at presentation ranged from 4 to 18 years, with most cases (87.2%) being > 5 years and 12.9% of cases were <5years which contrasts with the developed world, where it is rare for under 5 years of age. The proportions (15–30%) observed in various developing countries was in line with our findings [23–27]. The discrepancy for ages may be due to the high burden of infectious diseases in developing regions which can be compared to a particular distribution of histologic subtypes as discussed later.

In this cohort, male to female ratio was 3.4: 1. Male predominance in PHL was reported by many authors in developing countries [1, 25, 26, 28, 29]. The causes for gender distribution are not entirely understood. Research suggested that male children are more vulnerable to infections including EBV, and that social-cultural factors contribute to less care given to female children. In addition, in less economically developed populations of Asia and Africa, the initial illness incidence peak occurs among boys in early childhood rather than young adulthood, implying that PHL risk may relate to very early infection in these countries. The link between PHL and early infection is supported by the correlation of PHL risk with lower socioeconomic class markers such as lower parental income and increased housing density, both of which promote premature exposure to common infections [30].

One or more of the B-symptoms at diagnosis were noted in one-third of patients (34.3%), which is in line with previous studies from Egypt and India with 34%,39% and 32% respectively with comparable histologic subtype and staging of disease [23, 25, 31], However this result was higher than in studies done from Korea(14.8% [5] and lower than studies conducted in Bangladesh (59.6%) and South Africa (64%) [32, 33]. The discrepancies may be due to differences in histological subtype, stage of disease at presentation but detecting and documenting B symptoms may not be easy nor specific.

Majority of the cases (65.7%) were diagnosis by excisional biopsy whereas about one -fourth (27.1%) of cases were diagnosed using FNAC. Neck, chest and abdominopelvic CT-scan were done for all cases.

In our study, Nodular sclerosis was the commonest histologic subtype (34.3%) followed by mixed cellularity (21.4%), which is consistent with other studies done from low-income countries [23, 27, 33, 34]. On the other hand, mixed cellularity is the prevalent histologic subtype in low-income countries [25, 32, 35] and this discrepancy may be due to geographic difference in rate of infection in relation to EBV. Multiple etiologies for PHL have been proposed based on the distribution of patients across age, gender, geography, and socioeconomic status rather than a single disease. In underdeveloped nations, the mixed cellularity histological subtype is most common in young children, especially males, and typically manifests as advanced disease. Studies indicated a link between infectious mononucleosis and subsequent Epstein-Barr virus (EBV)-positive PHL [36–38]. In developed countries, childhood Hodgkin’s lymphoma primarily affects older children with the nodular sclerosis histological subtype. This may be due to delayed exposure to common infectious agents, as males are more susceptible to viral and bacterial infections in childhood, particularly during the first five years of life [39, 40].

In our findings, presentations with advanced stages of disease (stage III, IV) were noted in about 47% of patients. In Western countries, 75% of newly diagnosed patients had early disease at presentation (stage I–II) [24, 41].In most low-income settings, however, more than half of the patients have advanced disease at presentation (stage III–IV), perhaps because of delayed diagnosis and referral [38, 42] and our findings are in line with these studies.

About 68.6% (48) of patients completed their planned treatment. The most used chemotherapy regimen was ABVE-PC. Radiotherapy was administered for only 10% [7] patients and used only for those taking the ABVE-PC regimen. This regimen was almost in line with most protocols used in developing countries as intensive first line chemotherapies where radiotherapy was not readily available in most centers [5, 31, 43].

PHL is an example of childhood cancer, which can be cured in over 90% of cases with reasonable burden of late effects [44, 45]. The 3-years OS and EFS of our cohort were 98.6% and 85.7%, respectively. In Egypt reported OS of 96.6% and EFS of 84.7% while in Turkey reported OS of 96% and EFS of 72% [25, 27]. The 3-year EFS in our study was significantly lower in cases with advanced stages (III and IV) as compared with stage I and II, log rank *P* = 0.01. Similar results were reported in Egypt, and India [25, 46]. In addition, we observed that patient Age, Sex, histology, chemotherapy regimen and risk strata were not statistically associated with OS and EFS.

In our cohort, there was no advanced therapeutic follow up as well as diagnostic modalities like PET-FDG in which metabolic disease activity could have been better categorized and the information regarding number of cases excluded in this study was not available. Rate of treatment abandonment in our cohort was 31% affecting the cure rate and survival of PHL interpretation as abandoned cases were censored. The commonest causes of treatment abandonment in LMICs are poor awareness about cure, no health insurance, treatment related toxicities and progression/relapse of disease. The abandonment rate was comparable to other studies done in including Gondar pediatric cancer treatment center (39%) [47].Our study is the first of its kind in PHL treatment outcome assessment in the country so that protocols of treatment being used in the region can be tailored based on this study to meet the WHO’s target of survival by 2030.

## Conclusion

The survival functions in our studies are acceptable with the ABVE-PC and ABVD chemotherapy regimen-based therapies with risk-based radiation therapy added. The treatment outcome in our cohort surpassed the World Health Organization childhood indexed cancers treatment target by 2030 aiming 60% and above globally. In our cohort, FNAC had been used as diagnostic modality of PHL in substantial cases and Immunohistochemistry was almost never practiced for diagnosis. The study also showed that treatment abandonment remains the major challenge for cure in PHL in children affecting both cure and result interpretations (31%).

### Recommendations

Based on our recent study, the treatment protocol used in our cohort surpassed the WHO target of 60%. The diagnostic modality needs to be improved as FNAC is still being used to confirm the diagnosis as well as Immunohistochemistry needs to be incorporated for diagnosis. PET-FDG still is not being practiced for diagnostic as well as therapeutic follow-ups. We recommend researchers to study treatment abandonment and its contributing factors to address preventable causes.

## Data Availability

We confirm that all the data for this manuscript is available and can be shared upon request and if someone wants to request the data from this study it is directed at the first author (DTW).

## Abbreviations

PHL: Pediatric Hodgkin Lymphoma
ABVD: Adriamycin, Bleomycin, Vincristine and Dacarbazine
ABVE: Adriamycin, Bleomycin, Vincristine and Etoposide
PC: Prednisolone and Cyclophosphamide
EBV: Epstein Bar Virus
EFS: Event Free Survival
HL: Hodgkin Lymphoma
LMIC: Low- and Middle-Income Countries
OS: Overall Survival
RS: Reed Sternberg cells
